# Effects of Repetition Learning on Associative Recognition Over Time: Role of the Hippocampus and Prefrontal Cortex

**DOI:** 10.3389/fnhum.2018.00277

**Published:** 2018-07-11

**Authors:** Lexia Zhan, Dingrong Guo, Gang Chen, Jiongjiong Yang

**Affiliations:** ^1^School of Psychological and Cognitive Sciences, Beijing Key Laboratory of Behavior and Mental Health, Peking University, Beijing, China; ^2^Scientific and Statistical Computing Core, National Institute of Mental Health (NIMH), National Institutes of Health (NIH), Bethesda, MD, United States

**Keywords:** associative memory, repetition learning, consolidation, MTL, PFC

## Abstract

When stimuli are learned by repetition, they are remembered better and retained for a longer time. However, current findings are lacking as to whether the medial temporal lobe (MTL) and cortical regions are involved in the learning effect when subjects retrieve associative memory, and whether their activations differentially change over time due to learning experience. To address these issues, we designed an fMRI experiment in which face-scene pairs were learned once (L1) or six times (L6). Subjects learned the pairs at four retention intervals, 30-min, 1-day, 1-week and 1-month, after which they finished an associative recognition task in the scanner. The results showed that compared to learning once, learning six times led to stronger activation in the hippocampus, but weaker activation in the perirhinal cortex (PRC) as well as anterior ventrolateral prefrontal cortex (vLPFC). In addition, the hippocampal activation was positively correlated with that of the parahippocampal place area (PPA) and negatively correlated with that of the vLPFC when the L6 group was compared to the L1 group. The hippocampal activation decreased over time after L1 but remained stable after L6. These results clarified how the hippocampus and cortical regions interacted to support associative memory after different learning experiences.

## Introduction

It is well known that after repetition learning, memory performance can be enhanced and maintained for a long time (i.e., learning effect; Ebbinghaus, 1964). Less is known, however, about the brain mechanisms for the learning effect. Repetition suppression and repetition enhancement during memory encoding and retrieval are two phenomena that were reported in previous studies. During encoding, multiple learning leads to decreased activation in stimulus-related cortical regions and the hippocampus when compared to learning once (for reviews, Grill-Spector et al., 2006; Segaert et al., 2013). The repetition suppression in the hippocampus is confirmed when the single stimuli (e.g., pictures, Suzuki et al., 2011; Manelis et al., 2013) and stimulus associations are repeatedly presented (e.g., face-name pairs, Rand-Giovannetti et al., 2006; Vannini et al., 2013; face-scene pairs, Kremers et al., 2014 and object pairs, Zeithamova et al., 2016). During retrieval, studies which focus on implicit retrieval suggested that the hippocampal activation increases when subjects retrieve repeated items (vs. new items) by explicit strategy (e.g., Schacter and Buckner, 1998; for reviews, see Segaert et al., 2013; Kim, 2017), but those studies did not directly manipulate retrieval processes to explore the role of the hippocampus for the learning effect.

Only a few studies explored the effect of repetition learning on explicit retrieval (e.g., Heckers et al., 2002; Johnson et al., 2008; Kompus et al., 2009; Suzuki et al., 2011; Reagh et al., 2017), but obtained inconsistent results, especially in the hippocampus. For example, Heckers et al. (2002) revealed by PET that after words were repeated four times, recalling them led to increased activation in the left anterior hippocampus, and recalling one-presentation words led to increased activation in the bilateral dorsolateral prefrontal cortex (dLPFC) and right parahippocampal cortex (PHC). However, Reagh et al. (2017) showed that the hippocampal activation was weaker during retrieval after multiple rounds of learning than after learning once; in their study, subjects encoded pictures once or three times, after which they performed an old/new recognition task for the same, similar and new pictures. Note that one difference between the studies of Heckers et al. (2002) and Reagh et al. (2017) was that recall and recognition tasks were used separately. As suggested by previous findings, during the recall task, more detailed information is retrieved (Staresina and Davachi, 2006), and more recollection process is required than during the recognition task (Eichenbaum et al., 2007; Davachi and Dobbins, 2008). In contrast, the familiarity process is more involved during the recognition (vs. recall) task. Thus, these inconsistent findings may reflect different memory representations and processes underlying the task manipulation.

Behavioral studies have shown that after subjects learned words and word pairs for three or six times, their item and associative recognition performance increased significantly. In addition, both recollection and familiarity processes contributed to the learning effect (e.g., Barber et al., 2008; Yang et al., 2016). The higher recollection contribution to the repetition learning was shown from 10-min to 1-month intervals (Yang et al., 2016). As the hippocampal activation is associated with processing detailed information and the recollection contribution, the learning effect is more likely to manifest repetition enhancement rather than suppression during explicit retrieval. It is necessary to explore this issue when subjects retrieve detailed information during a recognition task.

A related issue is whether brain activation for different learning experiences changes in a similar way over time. Behavioral studies have shown that memory performance decayed more slowly after subjects learned stimuli several times (vs. learning the stimuli once), especially when the retention interval was within 1 week (e.g., Ebbinghaus, 1964; Yang et al., 2016). However, few neuroimaging studies focus on this issue. In some fMRI studies, the factor of learning experience is often mixed with that of retention interval, which ambiguates the interpretation of the results. For example, to compare recent and remote memories, stimuli for remote memory are sometimes learned for multiple times in order to obtain enough number of trials for analysis, but those for recent memory are learned once to exclude behavioral ceiling effect (e.g., Stark and Squire, 2000; Takashima et al., 2009; Yamashita et al., 2009). Thus, the difference in recent and remote memories is influenced by both the age of memory and learning experience.

Theories on memory consolidation have different predictions for the effect of repetitive learning on the hippocampus activation over time. According to the Standard Consolidation Theory (SCT, Squire and Bayley, 2007), repeated memories are more prone to becoming independent of the hippocampus and relying on the neocortex such as posterior perceptual regions (e.g., Takashima et al., 2009; Nieuwenhuis et al., 2012). Thus, with the passage of time, hippocampal activation should decrease more quickly after repetition learning (vs. learning the stimuli once), diminishing the learning effect in the hippocampus at longer intervals. On the other hand, based on the Multiple Trace Theory and Transformation Trace Theory (MTT and TTT; Moscovitch et al., 2006, 2016; Winocur and Moscovitch, 2011), the nature of memory representation determines whether memories depend on the hippocampus for their duration or whether they depend on the neocortex with time. Specifically, because repetition enhances the relational associations between items (e.g., Yang et al., 2016), as long as the associated pairs are remembered, those relational associations are preserved, predicting relatively sustained hippocampal activation across time.

In addition to the hippocampus, we are also interested in whether the repetition learning modulates the connectivity between the hippocampus and other regions over time. Previous studies have confirmed the interaction between the hippocampus and neocortical regions during explicit retrieval when stimuli are learned once (Köhler et al., 1998; Maguire, 2001). For a review, see Buckner and Wheeler, 2001; Simons and Spiers, 2003). There are strong anatomical connections between the hippocampus and the prefrontal cortex (PFC, Simons and Spiers, 2003), between the hippocampus and posterior cortical regions. The PFC is involved in searching, monitoring, inhibition and evaluation of relevant information at retrieval, and the posterior regions is involved in processing stimulus-related information (Simons and Spiers, 2003; Moscovitch et al., 2016; Eichenbaum, 2017). However, these processes may vary for the learning experience. Compared to learning once, repetition learning requires less executive controls (Heckers et al., 2002; Kompus et al., 2009) and more stimulus-related processing, so it is possible that the interaction between the hippocampus and PFC decreases, and the interaction between the hippocampus and stimulus-related regions increases, but empirical evidences are lacking.

In summary, here we focus on the issues of how learning experiences modulate brain activations and their connectivity with the passage of time. Clarifying these issues is important in that it helps elucidate how the neural basis of memory representation changes by learning experience. In this study, subjects learned face-scene pairs either once or six times during encoding. After 1-month, 1-week, 1-day and 30-min intervals, they were scanned when they performed an associative recognition task. We chose face-scene pairs as material because faces and scenes have definite and focal activation in the posterior regions (i.e., fusiform face area, FFA; posterior place area, PPA), which has an advantage to explore the relations between their activations and the hippocampus over time. Because there are different predictions on the effects of learning experience and retention interval on various brain regions, we focused on the hippocampus, the posterior regions (i.e., FFA and PPA) that are specific to faces and scenes, and the lateral PFC. The effects of learning and the retention interval were analyzed for these regions and their interactions.

Based on previous studies, we hypothesize that as repetition learning increases memory for detailed representations by recollection process (e.g., Yang et al., 2016), the associations studied six times are dependent on the hippocampus and remained stable over time; whereas associations studied only once are likely to lose their detailed representations and become dependent on extra-hippocampal and, likely, neocortical structures with time. Consequently, memory representation would be less likely to depend on the lateral PFC and the interaction of the lateral PFC-hippocampus. In contrast, as fewer details are remembered after learning the stimuli once, top-down control from the lateral PFC is obligatory to successfully retrieve the associative information, which increases the interaction of the lateral PFC-hippocampus.

## Materials and Methods

### Subjects

Thirty-seven right-handed subjects (16 males) with a mean age of 21.95 ± 2.56 years participated in the study. Among them, 20 subjects were randomly assigned to group L6, and the remaining 17 subjects were assigned to group L1. Five subjects’ fMRI data (4 subjects for L1 and 1 subject for L6) were excluded due to the excessive head motion during scanning (3 subjects), abnormal brain structures (1 subject) and low behavioral performance (1 subject). In the end, 16 subjects’ data in each group were entered into the data analysis. All subjects were native Chinese speakers and gave written informed consent in accordance with procedures and protocols approved by the department review board of Peking University.

### Materials

One within-subjects factor (retention interval: 20-min, 1-day, 1-week, 1-month) and one between-subjects factor (learning: once (L1), 6 times (L6)) were included in the study. Two hundred face-scene pairs were used as stimuli with 200 famous faces and 200 famous/familiar scenes selected based on various rating scores (see below). The famous faces included celebrities from inside or outside of China in sports, entertainment, academics, politics and industry. The familiar scenes included natural places of interest, buildings and indoor/outdoor pictures. The faces had moderate level of familiarity (4.60 ± 0.90), valence (5.72 ± 0.50) and arousal (4.82 ± 0.70) with no particular facial expression. The ratio of male vs. female was 7:3 and that of faces from inside vs. outside of China was 4:1. The famous faces were from different periods of time (1995–2012: 65%, 1980–1994: 20%, 1949–1980: 5.5%, 1900–1948: 8%, before 1900: 1.5%). The scenes had a moderate level of familiarity (4.92 ± 0.6) and complexity (3.70 ± 0.40).

The rating tests included two stages. First, 23 subjects (11 males, 21.70 ± 1.90 years) rated 450 faces for their familiarity, emotional valence and arousal and 450 scenes for their familiarity and complexity. All the ratings ranged from 1 (lowest) to 7 (highest). Two-hundred and fifty faces and 250 scenes were selected per the rating scores. Then, the faces and scenes were randomly combined to form 250 unrelated pairs. Second, another 11 subjects (4 males, 22.35 ± 1.27 years) rated the relatedness of the face and scene in each pair (1 was unrelated, 5 was related). The mean score of the final 200 pairs was 1.43 ± 0.28.

The 200 face-scene pairs were randomly assigned to 10 sets to be used as stimuli over four retention intervals (30-min, 1-day, 1-week and 1-month) and two pair types (old, recombined), resulting in 20 pairs per subset. The two sets of ten were used in the L1 group for testing during the 1-week and 1-month intervals to avoid the problem of low number of trials in the L1 group (i.e., 30 pairs for old, and 30 for recombined at 1-week and 1-month intervals). The 10 sets were matched in familiarity, complexity, valence, arousal and pair relatedness (all *F*’s < 1, *p’*s > 0.5). They were also matched in the face ratio of gender, age, profession, and perceptual features (all *F*’s < 1, *p’*s > 0.5). These 10 sets were also counterbalanced across subjects so that each set had an equal chance to be used in each condition.

### Procedure

**Figure 1:**
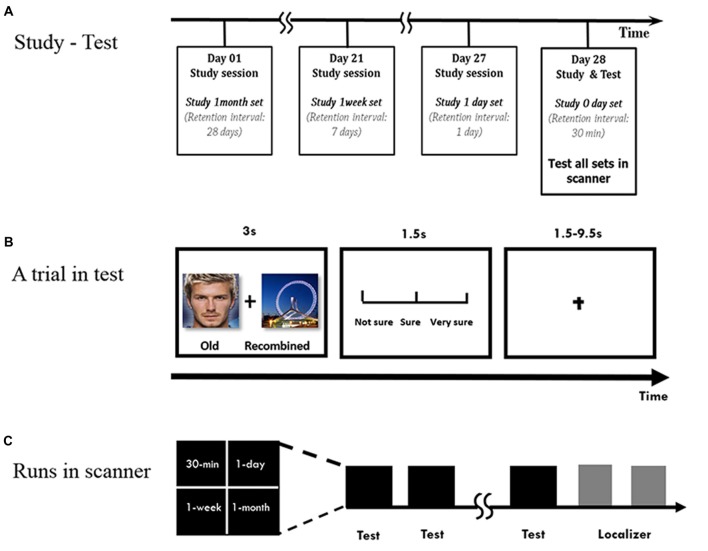
Experimental procedure. **(A)** Overview of the study-test procedure. **(B)** Within a run to test a face-scene association, each pair was presented for 3 s. Subjects were asked to make an old/new judgment, followed by the confidence rating. Chinese words are replaced by English words for illustration purpose. **(C)** Runs in scanner.

The subjects learned different sets of pairs at four retention intervals, with the last study session arranged 30 min before the test phase in the scanner (Figure 1A). During each study phase, the subjects learned the face-scene pairs. In each trial, a pair was presented at the center of the test screen for 5 s, during which the subjects performed one of two tasks, sentence making or imagination, in order to combine the unrelated face and scene. Then they were asked to determine the vividness of the sentence or imagination they made. The subjects in the L1 group performed the imagination task only. In contrast, because the subjects in the L6 group had to learn the pairs six times, both tasks were adopted alternately every other time. We used this manipulation for two reasons. One was to mimic repeated learning in real life situations, in which repeated learning is not simple repetitions but occurs with different contexts. The other was to reduce the effects of expectation and fatigue that would diminish the encoding efficiency and reduce recognition performance (Reagh and Yassa, 2014). All the pairs in the L6 group were presented in a six-round manner. The pairs were presented in a random order in both groups, and the random orders were different when they were presented six times in the L6 group.

During the test phase, the subjects were scanned while they performed the associative recognition task (Figure 1B). The old or recombined pairs were randomly presented for 3 s, and the subjects were instructed to determine whether they had seen the pairs during the study phase, followed by a confidence rating (from unsure to sure on a scale of 1–3) for 1.5 s. The inter-trial interval was adjusted in the event-related design to an average of 5.5 s (range: 1.5–9.5 s). The subjects had the chance to practice before the formal study and test phases.

The face-scene pairs were pseudo-randomly mixed in different runs during the test phase (Figure 1C). For the L6 group, there were four runs, with 10 pairs in each run per retention interval, half of which were old pairs and the other half were recombined. For the L1 group, because 20 more pairs for the 1-week and 1-month intervals were included separately, there were five runs with 8 pairs for 30-min and 1-day intervals and 12 pairs for the 1-week and 1-month intervals in each run, half of which were old pairs and the other half recombined. The pairs in each run was pseudo-randomly presented so that no more than three pairs in each condition were presented consecutively. The order of button presses and the order of the runs were counterbalanced across subjects.

After each study phase, the subjects were asked to perform a subtraction of 1000 minus 7 continuously to prevent the rehearsal of the stimuli. In addition, they were informed that it was not necessary to intentionally retrieve or forget the stimuli and were instructed to have sufficient sleep to ensure the memory consolidation (Diekelmann and Born, 2010). The average sleeping time after the different study phases was comparable across intervals: 7.5 ± 1.5 (1-day), 7.4 ± 0.7 (1-week) and 7.8 ± 0.7 (1-month).

To circumscribe the locations representing faces and scenes in the brain, a separate localizer session was performed for each subject (Epstein and Kanwisher, 1998) after the recognition test. Each face or scene was presented for 500 ms after a fixation of 500 ms. The subjects were asked to make a one-back identification task to judge whether the current picture was the same as the previous one. There were two runs for localization, with each run having three blocks for faces, three for scenes and four for fixations. For each face or scene block, there were 20 pictures, two of which were repeated for the identification task. Each run lasted 200 s. The stimuli in the two runs were the same, but the block order was different in the two runs. The faces and scenes were also different from the fMRI stimuli but matched for various features (e.g., familiarity, arousal and time period).

### MRI Acquisition

The MRI data were collected on a Siemens Trio 3T scanner. Functional data were acquired using a gradient echo-planar imaging (EPI) sequence. Anatomical data were acquired using a high-resolution MP-RAGE sequence (TR = 920 ms, TE = 9.2 ms, flip angle = 37°, FOV = 22 cm, 256 × 256 matrix, resolution = 1 × 1 × 1.3 mm^3^) before functional scanning. The parameters used for the EPI sequence were TR = 2 s, TE = 30 ms, flip angle = 90°, FOV = 19.2 cm, matrix = 64 × 64, slice = 33, resolution = 3 × 3 × 3 mm^3^. The test phase was performed first and followed by the localizer task and the anatomical scan.

### Image Analysis

The AFNI software program was used to pre-process the imaging data and for the statistical analysis (Cox, 1996). The EPI volumes were registered, smoothed with a FWHM of 4 mm, and scaled to a voxel-wise mean of 100. They were then warped into the standard space of the Talairach and Tournoux (1988) atlas before the individual subject analysis (3dDeconvolve), in which a time window of about 12 TRs (24 s) was determined to capture the BOLD response of each stimulus with a presumed hemodynamic response function. The trials were labeled as Hit, false alarm (FA), correct rejection (CR) and Miss. Altogether, 16 regressors of interest (4 retention intervals by 4 types of trials) and 6 regressors of no interest (motion parameters) were applied, and the estimated β weights indicated the BOLD response amplitude for each condition. Anatomical volumes were also warped into the standard stereotaxic space of the Talairach and Tournoux (1988).

The group analysis included two aspects. First, to determine the difference between the experimental conditions in the whole brain, the voxel-wise mixed-effects analyses of variance (ANOVA) was performed on the Hit trials (i.e., old trials that were correctly judged as “old”) from the individual analysis, with learning group and retention interval as fixed-effects factors and the subject as a random-effects factor. The effects of learning and retention interval and their interactions were reported. We also analyzed the Hit trials with “sure” and “very sure” ratings, and the results were similar to those with all Hit trials. To get more trials to the model and to achieve higher detection power, we reported the results with all Hit trials in this article.

In particular, we focused on the ANOVA activation in the predetermined regions, i.e., MTL and stimulus-related regions based on the hypotheses. The MTL subregions were manually drawn in the standard space for each subject (Insausti et al., 1998; Pruessner et al., 2000, 2002; Frankó et al., 2014), including the hippocampus head/body (i.e., anterior), hippocampus tail (i.e., posterior), perirhinal cortex (PRC)/EC and PHC. In brief, the MPRAGE coronal plane was used to segment the subregions of the MTL. The anterior border of the hippocampus was usually found in the most rostral of the lateral ventricle, and the end of the hippocampus was defined as the disappearance of the ovoid gray matter medially to the lateral ventricle. The anterior border of the PRC was defined as the most anterior slice in which the collateral sulcus was visible (Ritchey et al., 2015), and its most posterior slice was defined as 3 mm after the gyrus intralimbicus disappeared. As it is hard to dissociate the PRC from EC, we combined them as one ROI. The PRC was replaced caudally by the anterior of the PHC. The posterior extent of the PHC was defined as the anterior limit of the parieto-occipital fissure. The segmentation of the MTL was co-registered to standard space and averaged across subjects.

In addition, in the localizer task, we identified the bilateral parahippocampal place area (PPA; left: −28, −49, −10; right: 29, −49, −13) and the right FFA (44, −43, −19), which showed stronger activation for scenes (vs. faces) and faces (vs. scenes), separately. Monte Carlo simulations were used to correct for multiple comparisons and to determine the minimum cluster size for the corrected *P* of 0.05 in cortical regions (volume = 1890 mm^3^). Small volume correction (SVC) was used for the MTL subregions (volume = 810 mm^3^), PPA and FFA (volume = 648 mm^3^). Brain activation was reported at the level of *p* < 0.05 corrected (two-tailed).

Furthermore, to explore whether the connectivity between the hippocampus and neocortical regions, including the PPA/FFA and prefrontal cortex, differed when the subjects learned the pairs once or six times, psychophysical interaction (PPI, Friston et al., 1997) analysis was applied. We used three different PPI seeds, i.e., a sphere with a radius of 5 mm in the bilateral PPA ROIs and the bilateral ventrolateral prefrontal cortex (vLPFC) activation from the group effect. To perform the PPI analysis, the time series were extracted from the seed regions under each condition for each subject and deconvolved with the HRF to obtain the corresponding neural activities (Gitelman et al., 2003). The interaction regressors and the seed time-series regressor were then entered into the original univariate design matrix. Finally, the β value associated with each interaction regressor was used for the ANOVA analysis (McLaren et al., 2012; Cisler et al., 2014; two-tailed, *p* < 0.05 corrected).

## Results

### Behavioral Results

**Figure 2:**
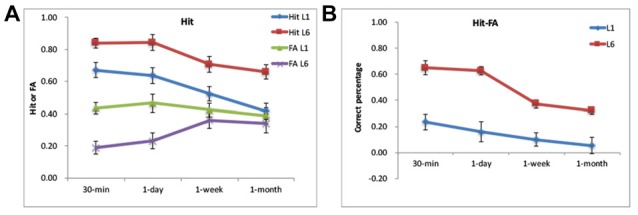
Behavorial results. **(A)** The Hit and false alarm (FA) rates for the L1 and L6 groups. **(B)** The Hit-FA performance for the L1 and L6 groups.

The scores for Hit rate, FA rate, corrected recognition (Hit-FA) and reaction times (RTs) were analyzed with a repeated-measures ANOVA with the retention interval as a within-subject factor and learning (or group) as a between-subjects factor. For Hit rate, L6 led to a higher Hit rate than L1 (0.75 ± 0.17 vs. 0.55 ± 0.14, *F*_(1,30)_ = 13.31, *p* < 0.001) at different retention intervals (Figure 2A). This enabled us having enough number of trials for fMRI analysis. The accuracy decreased over time for both groups (*F*_(3,90)_ = 30.59, *p* < 0.001). A further analysis showed that the decrease occurred especially from 1-day to 1-month. There was no significant difference between the 30-min and 1-day intervals in both groups (*p =* 1.0). The interaction between the retention interval and learning was not significant (*F*_(3,90)_ = 0.44, *p =* 0.72). The analysis of Hit rate with high confidence provided similar results. Subjects presented “sure” and “very sure” ratings out of the correct response in over 60% for each condition (0.69 ± 0.17 for L1 and 0.88 ± 0.09 for L6). There were more number of “sure” and “very sure” responses for L6 than L1, *F*_(1,30)_ = 15.21, *p* < 0.001. The number of “sure” and “very sure” responses decreased over time, *F*_(3,90)_ = 12.88, *p* < 0.001, especially from 1-day to 1-week (*p* = 0.02).

For FA rate, L6 led to lower FA rates than L1 (0.29 ± 0.18 vs. 0.42 ± 0.17, *F*_(1,30)_ = 6.45, *p =* 0.02). There was a significant interaction between retention interval and learning (*F*_(3,90)_ = 11.30, *p* < 0.001). This was because the FA rate was higher for L1 than L6 for 30-min and 1-day (*p’*s < 0.01) but was comparable between the two groups for the longer retention intervals (*p*’s > 0.5). The FA rate increased from 1-day to 1-week for the L6 group (*p* < 0.01) but increased from 1-day to 1-month for the L1 group (*p* < 0.01).

For the corrected recognition, the accuracy decreased over time for both groups (*F*_(3,90)_ = 26.72, *p* < 0.001). The memory performance in L6 was higher than in L1 (0.48 ± 0.18 vs. 0.13 ± 0.10) across retention intervals, *F*_(1,30)_ = 48.21, *p* < 0.001; Figure 2B). The interaction between the retention interval and learning was significant (*F*_(3,90)_ = 5.33, *p =* 0.002), but the group difference occurred at each retention intervals (*p*’s < 0.001). Further analysis showed that memory decay occurred specifically between 1-day to 1-week for the L6 group (*p*’s < 0.05), whereas decreased linearly for the L1 group (*p*’s < 0.05).

L6 had comparable RTs to L1 (*F*_(1,30)_ = 1.92, *p =* 0.18). There was no significant effect of retention interval or its interaction by learning group (*p*’s > 0.05).

### Learning Effect in the Hippocampus, PRC/EC and PFC

**Figure 3:**
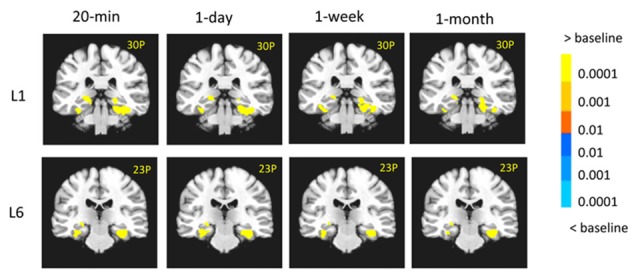
The medial temporal lobe (MTL) activity in each condition. The MTL was activated at different retention intervals for both L1 and L6 groups. The left is on the left side.

We first looked at whether the MTL, especially the hippocampus, was activated under each condition. Figure 3 shows that, compared to the fixation, the MTL was activated at different retention intervals for both L1 and L6 groups (*p’*s < 0.0001). It confirmed that the hippocampus and other MTL regions were involved in retrieving associative memory irrespective learning experience and retention interval.

**Figure 4:**
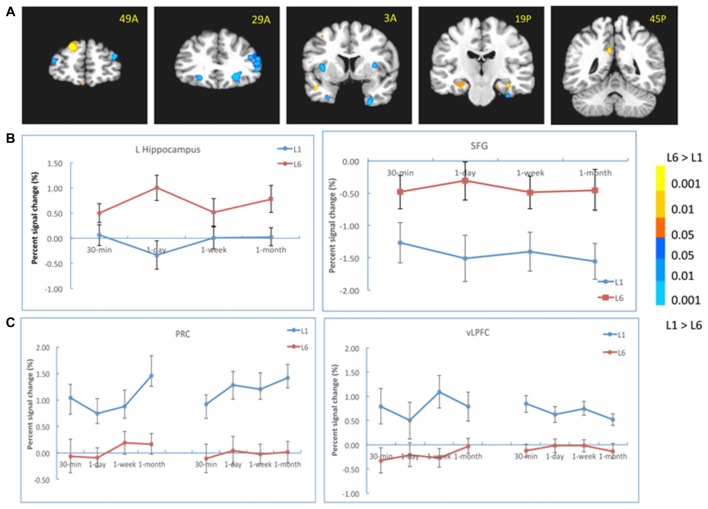
Results of the learning effect. **(A)** The left hippocampus, the posterior cingulate cortex (PCC) and the superior frontal cortex (SFG) showed stronger activation for the L6 than for the L1 group, whereas the bilateral perirhinal cortex (PRC), the right inferior prefrontal cortex (IFG), bilateral ventrolateral prefrontal cortex (vLPFC) and bilateral insula/thalamus showed stronger activation for the L1 than for the L6 group. **(B)** The activation of the hippocampus and SFG at each retention interval. **(C)** The activation of the PRC and vLPFC at each retention interval. The color bar represents the contrast between L1 and L6. The left is on the left side for each brain slice.

When the two groups were directly compared, the results showed that there were stronger activations in the left hippocampus (−29, −20, −16, *t*_(30)_ = 2.65, *p* < 0.05), the posterior cingulate cortex (PCC, 2, −62, 27, *t*_(30)_ = 3.23, *p* < 0.005) and the superior frontal cortex (SFG, −17, 50, 36, *t*_(30)_ = 4.45, *p* < 10^−4^) for L6 compared to L1. In contrast, there were stronger activations in the bilateral PRC (left: −26, −2, −34, *t*_(30)_ = 3.24, *p* < 0.01; right: 26, 5, −40, *t*_(30)_ = 3.88, *p* < 0.005), the right inferior prefrontal cortex (IFG), the bilateral vLPFC (left: −23, 29, −7, *t*_(30)_ = 3.42, *p* < 0.001; right: 23, 30, −5, *t*_(30)_ = 3.92, *p* < 0.001) and the bilateral insula/thalamus for L1 compared to L6 (left: −32, 38, 15, *t*_(30)_ = 3.58, *p* < 0.005; right: 53, 26, 3, *t*_(30)_ = 3.83, *p* < 0.005; Figure 4A). The bilateral PPA and right FFA did not have a significant learning effect but showed a significant interaction between learning group and retention interval. Note that the right PPA showed stronger activation for L6 than L1 (23, −65, −4, *t*_(30)_ = 3.17, *p* < 0.005) when Hit-FA was the dependent measure, and this pattern appeared at different intervals except at 30-min. This was mainly because the PPA activity for the FA trials increased over time, leading to increased activation in the right PPA from 1-day to 1-month.

We also analyzed the learning effect in these regions at each retention interval. The results showed that the left hippocampal activation was stronger for L6 than L1 at 1-day (−26, −17, −13, *t*_(30)_ = 3.56, *p* < 0.005) and 1-month (−26, −38, 6, *t*_(30)_ = 3.41, *p* < 0.005) intervals (Figure 4B). Its activation was comparable between the two groups at 30-min interval. The activations in the PCC and SFG were stronger for L6 than L1 at each retention interval. There were weaker activations in the bilateral PRC and vLPFC for L6 compared to L1 at each retention interval (Figure 4C). These results suggested that repetition learning modulates the involvement of the hippocampus and other MTL subregions in memory retention and retrieval.

### Time Effect in the MTL Differed by Learning Experience

A significant effect of retention interval was found in the MTL and other brain regions. Specifically, the bilateral hippocampus (left: −23, −8, −16, *F*_(3,90)_ = 4.77, *p* < 0.005; right: 23, −5, −19, *F*_(3,90)_ = 7.01, *p* < 10^−4^) and the left PRC (−20, 8, −28, *F*_(3,90)_ = 6.26, *p* < 0.001) showed significant time-related changes. Other regions included the PCC, inferior parietal cortex, middle frontal cortex, and the right vLPFC (23, 50, 3, *F*_(3,90)_ = 5.84, *p* < 0.001). The bilateral PPA and the right FFA did not show significant time effect.

**Figure 5:**
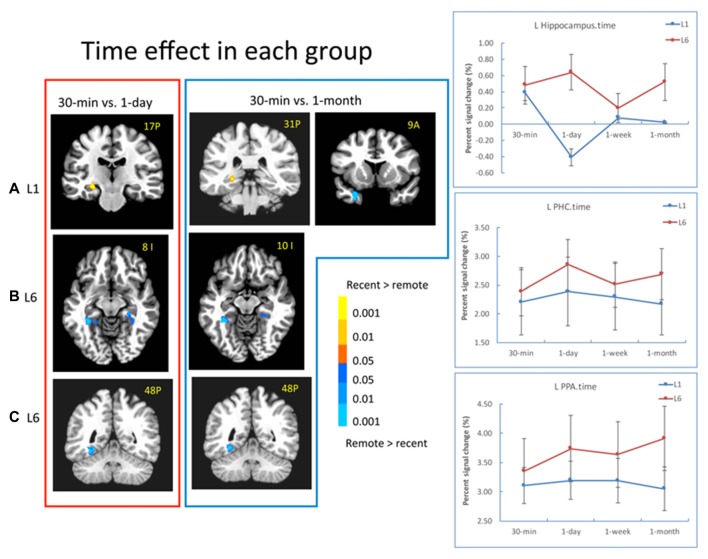
Results of the time effect. **(A)** For the L1 group, the hippocampal activation decreased from 30-min to 1-day, and 30-min to 1-month. The EC/PRC showed increased activation from 30-min to 1-month. **(B,C)** For the L6 group, the activation of the parahippocampal cortex (PHC) and parahippocampal place area (PPA) significantly increased when 30-min was compared with 1-day and 1-month intervals. The brain activation in the red frame represents the contrast of 30-min vs. 1-day, and that in the blue frame represents the contrast of 30-min vs. 1-month. The left is on the left side for each brain slice. The color bar represents the contrast between recent vs. remote intervals (e.g., 30-min vs. 1-day). The signal changes of the left hippocampus, PHC and PPA in the contrast of 30-min vs. 1-day (i.e., red frame) are plotted for each condition.

There was a significant interaction between retention interval and learning in the left hippocampus (−29, −35, 6, *F*_(3,90)_ = 6.97, *p* < 0.001), left EC/PRC (−32, 5, −16, *F*_(3,90)_ = 5.15, *p* < 0.005), left PHC/PPA (−41, −35, −13, *F*_(3,90)_ = 6.49, *p* < 0.001) and the bilateral vLPFC (left: −26, 32, −1, *F*_(3,90)_ = 5.62, *p* < 0.005; right: 35, 29, 3, *F*_(3,90)_ = 5.80, *p* < 0.005). For the L1 group, the activation in the left hippocampus decreased from 30 min to 1 day (−26, −17, −10, *t*_(15)_ = 4.21, *p* < 10^−4^) and from 30-min to 1-month (−23, −29, −4, *t*_(15)_ = 4.01, *p* < 10^−4^). The activation in the EC/PRC increased from 30 min to 1 month (−28, 11, −22, *t*_(15)_ = 2.83, *p* < 0.02; Figure 5A). In contrast, for the L6 group, the hippocampus activation did not show significant changes across retention intervals, but the activation of the PHC increased significantly from 30-min to 1-day (left: −17, −41, −1, *t*_(15)_ = 3.31, *p* < 0.005; right: 26, −32, −13, *t*_(15)_ = 5.16, *p* < 10^−4^) and to 1 month intervals (left: −31, −44, −10, *t*_(15)_ = 4.13, *p* < 0.001; right: 31, −26, −19, *t*_(15)_ = 3.70, *p* < 0.001; Figure 5B).

In addition to the MTL regions, in the L6 group, the left PPA activation increased significantly from 30-min to 1-day (−29, −65, −10, *t*_(15)_ = 4.96, *p* < 10^−4^), to 1-week (−32, −47, −13, *t*_(15)_ = 2.96, *p* < 0.01) and to 1 month (−32, −47, −10, *t*_(15)_ = 4.76, *p* < 10^−4^; Figure 5C). The signal changes of the left hippocampus, PHC and PPA for the contrast of 30-min vs. 1-day are plotted for each condition in Figure 5 (right panel). These results suggested that repetition learning not only leads to stable hippocampus activation over time, but also leads to increased activation in extra-hippocampus and neocortical regions with the passage of time.

### Correlation of Hippocampus-vLPFC and Hippocampus-PPA Differed by Learning Experience

**Figure 6:**
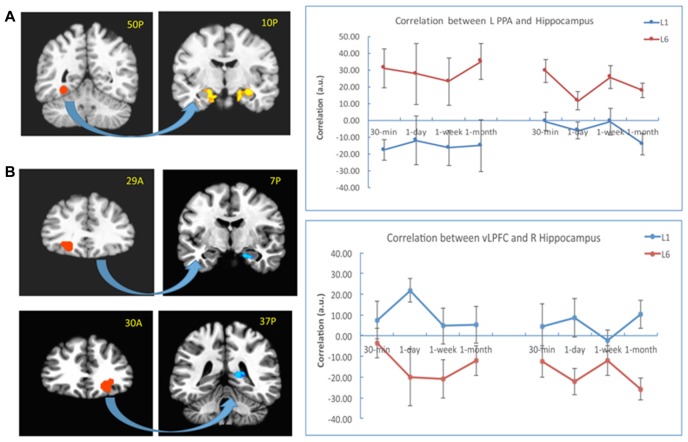
PPI results with the PPA and vLPFC as seeds. **(A)** The connectivity between the left PPA (as seed in the left) and the bilateral hippocampus (in the right) increased for the L6 (vs. L1) group. **(B)** The connectivity between the bilateral vLPFC (as seeds in the left) and the hippocampus (in the right) decreased for the L6 (vs. L1) group. The warm color represents increased connectivity for L6 vs. L1, and the cold color represents increased connectivity for L1 vs. L6. The left is on the left side for each brain slice.

To explore whether the correlation between the hippocampus and neocortical regions differed when the subjects learned the pairs once or six times, we first chose the bilateral PPA from the localizer as the seed. The results showed that the connectivity was stronger for the L6 group relative to the L1 group between the left PPA and the bilateral hippocampus (left: −11, −11, −19, *t*_(30)_ = 4.27, *p* < 10^−4^; right: 26, −29, −19, *t*_(30)_ = 4.08, *p* < 10^−4^), and between the right PPA and the left hippocampus (−25, −25, −10, *t*_(30)_ = 2.97, *p* < 0.01). This pattern was consistent across all retention intervals (Figure 6A).

We then chose the left and right vLPFC (from group effect) as seeds for the PPI analysis. The results showed that the connectivity between the left vLPFC and the right hippocampus (23, −11, −19, *t*_(30)_ = 5.02, *p* < 10^−4^) was stronger for the L1 (vs. L6) group. This pattern appeared for each retention interval except the 30-min interval. There was also stronger connectivity between the right vLPFC and the right hippocampus (17, −35, 3, *t*_ (30)_ = 2.99, *p* < 0.02) for the L1 (vs. L6) group. This pattern appeared for each retention interval except the 1-week interval (Figure 6B). There was no significant connectivity between the left vLPFC and posterior regions except that at the 30-min interval, their connectivity was stronger for the L6 than L1 group (23, −47, −7, *t*_(30)_ = 4.04, *p* < 10^−4^).

## Discussion

The objective of this study was to explore whether learning experience is associated with different brain activations in the MTL and neocortical regions, and how the activations change over time. The results show that, compared to learning once, learning six times led to stronger activations in the hippocampus, and weaker activations in the PRC and vLPFC. In addition, the hippocampal activation was positively correlated with that of the PPA, and negatively correlated with vLPFC for the L6 (vs. L1) group. Regarding the time change, the hippocampal activation decreased over time after L1 but increased over time after L6. These results showed how the hippocampus and cortical regions interacted with associative memory with the passage of time after different learning experience.

### Learning Effect in the Hippocampus and PRC/EC

One of the novel findings in this study was that MTL activation was dissociated with the learning effect. There was stronger activation in the left hippocampus, whereas weaker activation in the bilateral PRC/EC for the L6 than L1 group. This finding suggested that learning multiple times triggers both repetition enhancement and suppression in the MTL and confirmed the important role of the hippocampus in retrieving associative information after repetition learning.

The results reconciled the inconsistent results of the hippocampus activation in previous studies (e.g., Heckers et al., 2002; Johnson et al., 2008; Suzuki et al., 2011; Reagh et al., 2017), suggesting that the underlying process and retrieved information determine whether the repetition enhancement or suppression occurs in the hippocampus during explicit retrieval (Moscovitch et al., 2016). When recollection process is primarily involved and retrieving associations between faces and scenes are required, the hippocampus is involved and its activation is increased. The hippocampus is critical to encoding, consolidating and retrieving detailed and associative information (Mayes et al., 2007; Moscovitch et al., 2016). In addition, the hippocampus is more involved in the recollection process, whereas the PRC is more involved in the familiarity process in recognition memory (Brown and Aggleton, 2001; Eichenbaum et al., 2007; but see Squire et al., 2007). Previous studies have shown that repetition learning significantly increased the memory performance for detailed and associative information, and at the same time, increased the recollection contribution in associative memory (Barber et al., 2008; Yang et al., 2016). Therefore, the increased hippocampal activation after repetition learning is due to retrieving more detailed/associative information (i.e., the relations between the face and scene) and higher recollection contribution. Rat studies have also suggested that the hippocampus is important for contextual memory even after repetition learning (e.g., Lehmann et al., 2013). Note that the stronger hippocampus activation for L6 than L1 in the current study did not occur until the 1-day interval, suggesting that the learning effect in the hippocampus may need a consolidation process.

Different from the hippocampus, the bilateral PRC/EC showed weaker activation for the L6 than L1 group. This was consistent with previous findings that the PRC activation is decreased after repetition learning during explicit retrieval (Brown and Aggleton, 2001; Eichenbaum et al., 2007). The PRC is shown to be more involved in item familiarity than the recollection process (Eichenbaum et al., 2007; Ranganath and Ritchey, 2012). When familiarity (and/or implicit retrieval) is largely contributed in memory retrieval, the hippocampus activation does not change significantly. Montaldi et al. (2006) showed that when the subjects retrieved the scene memory, the PRC activation was modulated by the strength of familiarity: the higher the familiarity was, the lower the PRC activation was. The weaker activation in the PRC reflects that repetition triggers more selective responses. The PRC also supports associative memory by processing contextual episodic details that are able to be unitized as a single representation (Staresina and Davachi, 2006; Haskins et al., 2008). This typical repetition suppression (Grill-Spector et al., 2006; Segaert et al., 2013) shown in the PRC suggests that learning the face-scene pairs six times could establish a memory representation that is less based on item familiarity or rigid association.

### Time Effect in the Hippocampus and Other Regions

As for time change, our results showed that there were different patterns between L1 and L6. For the L1 group, the hippocampus activation decreased from 30-min to 1-day and from 30-min to 1-month. For the L6 group, the hippocampus activation remained relatively stable. In this study, the learning experience was controlled to compare the signal changes over different retention intervals (Bosshardt et al., 2005). The results clarified how repetition learning modulated memory consolidation and retrieval over time.

These results were consistent with the hypothesis based on the MTT/TTT (Moscovitch et al., 2006, 2016; Winocur and Moscovitch, 2011). The nature of memory representation determines the hippocampus involvement with the passage of time. As long as the associated pairs are remembered, hippocampal activations are sustained over time. In the L6 group, the relationship between the face and scene were successfully established after learning the stimuli six times, even at the early stage of memory consolidation; thus, the hippocampus activation remained stable during memory retrieval. In addition, the activation in the PHC and PPA increased over time when 1-day and 1-month were compared to 30-min after L6, suggesting that repetition learning enhances the processing related to the stimuli and contextual information. In contrast, in the L1 group, with the passage of time, associative memory may lose detailed information and become more familiarity-based. Both changes lead to decreased hippocampal involvement over time (Winocur and Moscovitch, 2011; Moscovitch et al., 2016). The latter mechanism is also supported by the increased activation in the PRC/EC over time for L1 in the current study.

Some previous studies have shown that the hippocampus activation decreased over time after repetition learning. For example, when subjects learned face-location pairs multiple times, the hippocampus activity decreased while the neocortical activity increased with consolidation (Takashima et al., 2009; Nieuwenhuis et al., 2012). It is worth noting that the locations in those studies were six positions on the screen, whereas our study and Bosshardt et al. (2005) used pairs of two different stimuli. Memory for face-location pairs are more likely to depend on schematic-based process (Tse et al., 2007), which can facilitate the transformation of memory representation from the hippocampus to the neocortex (van Kesteren et al., 2012). The associative memory of face-scene pairs and word pairs, on the other hand, relies more on detailed information than schematic information and is more hippocampus-dependent even after a long period of time.

### Learning Effect in the PFC

In addition to the MTL, the vLPFC showed a significant learning effect in the current study. Compared to L1, the regions in the PFC were less activated in L6, including the anterior vLPFC and inferior PFC. Our results suggested that learning experience modulates the involvement of the PFC in episodic retrieval. Repetition learning decreases the involvement of the prefrontal cortices, and their activation appears more important when learning the stimuli once.

Unlike item memory, the associative memory requires multiple processes (e.g., controlled cue specification and recollective monitoring) that depend on the prefrontal cortex (Dobbins et al., 2002). After learning once, the regions in the PFC are largely activated, including the anterior vLPFC and inferior PFC, which provide executive control to memory retrieval (e.g., Dobbins and Wagner, 2005; Dobbins and Han, 2006; Kompus et al., 2009). In a study by Badre et al. (2005), when the subjects were presented with a cue word and a set of target words, they chose one of the targets based on its semantic relationship with the cue. The results showed that the vLPFC was more activated when the strength of the cue-target associations was weak (e.g., candle-halo) than when they were strong (e.g., candle-flame), suggesting that the vLPFC is important in retrieving weak associative memories. In addition, the anterior vLPFC supports controlled retrieval processes to elaborate the cue used to probe the memory, thereby favoring memory for detailed information (Badre and Wagner, 2007).

As proposed by Shing et al. (2010), episodic memory builds on two interacting components, i.e., associative and strategic. Multiple learning increases the hippocampus involvement and hence enhances the associative component. For multiple learning, associations are built up for information from various sources; thus, during retrieval, the associative information could act as cues and does not need effortful searching of correct answers, which is mediated by significant control processing in the vLPFC. In contrast, the higher control processing or strategic component may become necessary in retrieving associative information after learning the stimuli once. When the bottom-up activation of relevant knowledge is diminished or insufficient to elicit the activation of target knowledge, the anterior vLPFC and other PFC regions provide top-down influences on the hippocampus and posterior cortical regions, facilitating the retrieval of memory-related information (Badre and Wagner, 2007).

We did not find significant vmPFC activation in the learning effect, although previous studies suggested its involvement in episodic encoding and retrieval (e.g., van Kesteren et al., 2012; Gilboa and Moscovitch, 2017; Liu et al., 2017). This is mainly because the lateral PFC and medial PFC are involved in different memory processes (Gilboa and Moscovitch, 2017). The vmPFC supports the updating of memory organizations and the assimilation of new memories into preexisting networks of knowledge (Hebscher and Gilboa, 2016; Moscovitch et al., 2016; Gilboa and Moscovitch, 2017).

### Brain Connectivity Related to the Hippocampus After Repetition Learning

Retrieving episodic associations relies on a distributed functional network between the MTL/hippocampus and PFC-mediated cognitive control processes (Buckner and Wheeler, 2001; Simons and Spiers, 2003; Wang and Morris, 2010). Our results suggested that there are two distinct brain networks related to learning experience. There was stronger connectivity between the hippocampus and PPA after L6 than L1 group, whereas stronger connectivity occurred between the hippocampus and vLPFC after L1 than L6 group. Thus, the learning experience not only modulates the activation of these regions, but also influences the connectivity between the hippocampus, prefrontal cortex and posterior regions during memory retrieval.

The results suggested that multiple learning may increase the connectivity between the hippocampus and neocortical regions during memory retrieval. The PPA processes and stores spatial-related information, and lesions in the region close to PPA lead to impaired scene processing (Epstein and Kanwisher, 1998). During retrieval, a rapid interaction between the cue and hippocampus occurs first, which in turn reactivates the neocortical traces bound to it (Moscovitch et al., 2016). These posterior neocortical components, in conjunction with the hippocampus, determine the local spatio-perceptual aspects of the experience (Buckner and Wheeler, 2001; Moscovitch et al., 2016). Thus, the stronger connectivity between the hippocampus and the PPA in L6 (than L1) enables the information from different sources to bind together and support the retrieval of scene-related associations after repetition learning.

On the other hand, stronger connectivity between the hippocampus and vLPFC in L1 may represent a top-down fashion control involving the MTL in processing the information that is relevant to retrieving associations. This aspect of our results suggested that the prefrontal cortex is more involved in retrieving associative memory after L1 than L6. The PFC operates on the information delivered to the hippocampus and on the output from it to make memory goal-directed (Simons and Spiers, 2003). When memories retain more gist or familiarity, they are more likely to become incorporated into a semantic network and acquire its properties (Moscovitch et al., 2016). Therefore, learning the stimuli once leads to greater demands on the processes of organization and strategic retrieval search (Simons and Spiers, 2003).

Our study also indicated that the connectivity between the PPA and hippocampus was active at four retention intervals, and the connectivity between the left vLPFC and hippocampus was present across four retention intervals except the 30-min interval. These results suggested that memory information becomes distributed across cortico-hippocampus networks at various stages of consolidation (Tambini et al., 2010; Vilberg and Davachi, 2013; Dudai et al., 2015). Once the connectivity is established, it remains stable until the 1-month interval.

### Learning Effect and Task Manipulation

In this study, we used the imagination task in the L1 group and both the imagination and sentence making tasks in the L6 group. One may argue that the learning effect was due to the additional task in the L6 group. However, we believe that this should not influence the results and interpretations. First, in the L6 group, the face-scene pairs were presented six times in six rounds. When the pairs were presented repeatedly with time spacing, the subjects more likely retrieved the previous memory trace and encoded the same pairs in different/changing contexts. Thus, variable encoding provides more ways to which the target information can be accessed in a later test (for reviews, see Cepeda et al., 2006; Gerbier and Toppino, 2015; Maddox, 2016). In other words, the memory representation is strengthened as well (LaRocque et al., 2013). Therefore, the difference in task types may influence the magnitude of the memory enhancement, but it should not influence the underlying mechanisms.

In addition, simply repeating stimuli is not sufficient to enhance memory performance (Reagh and Yassa, 2014), partly because the repeatedly presented pairs attract less attentional resources and are more likely transformed into semantic-like representations (Yassa and Reagh, 2013). Without encoding variability, the difference between L1 and L6 could also be explained as a strong vs. weak memory effect rather than the learning effect.

Furthermore, the task difference should not result in different patterns of brain activation in the MTL and PFC. The activations of the MTL and PFC have been observed when the encoding tasks are involved in making a sentence (e.g., Giovanello et al., 2004, 2009) and in forming a mental image (e.g., Lepage et al., 2003; Jackson and Schacter, 2004). As suggested by previous studies, subjects likely remember both task-relevant sources in addition to task-irrelevant sources (Uncapher et al., 2006; Song et al., 2011). Compared to stimulus features (e.g., item or association, related or unrelated association, number of contexts; Achim et al., 2007), the task performed by the subjects is only one source of the remembered items and associations, and should not change the activation pattern. In contrast, the type of information and type of process are the critical factors in determining the activation in the MTL and PFC (Moscovitch et al., 2016).

There are some potential limitations for this study. First, although the variable encoding strategy fits our purpose, we could not fully rule out the confound of different encoding tasks, which may bring some uncertainty regarding the data interpretations. Second, as we did not record the encoding performance, we were unable to analyze the difference in the encoding for the repetition effect. Third, we did not apply remember/know paradigm during the recognition task. The evidences of the recollection and familiarity processes due to repetition learning were only obtained from previous studies (e.g., Barber et al., 2008; Yang et al., 2016). Further studies could adopt the same encoding task and record the encoding performance to avoid the encoding confounds. The remember/know paradigm could be adopted to get direct relations between behavioral and brain activations. In addition, when stimuli are learned multiple times, they are more likely to be associated with emotional contexts, inducing different preference to the stimuli. Previous studies have shown that emotional context enhances memory by increasing the hippocampal activation and its connectivity with the amygdala (e.g., Erk et al., 2005; Smith et al., 2006; Takashima et al., 2016). It would be interesting to investigate in future studies whether the activation of the two hippocampo-cortical networks in L1 and L6 is related to the difference in emotional contexts and preference.

## Summary

The current study confirmed that the hippocampus was involved in associative memory retrieval regardless of learning experience and retention interval. More importantly, the learning experience modulated the associative memory by activating two distinct hippocampus-related brain networks. Multiple learning significantly increased activation in the hippocampus and the connectivity between the hippocampus and posterior regions and led to successful associative memory retrieval. In contrast, memory retrieval after L1 required executive control functions from the vLPFC, showing that L1 increased the activations in the vLPFC and PRC and the connectivity between the hippocampus and vLPFC. The activation of the hippocampus decreased over time after L1 but remained stable after L6. These results shed light on how distinct brain networks work in concert to support the retrieval of associative memories and how repetition learning influences subsequent memory over time.

## Author Contributions

LZ designed the experiments, performed the experiments and analyzed the data. DG and GC analyzed the data and wrote the article. JY conceived and designed the experiments, analyzed the data and wrote the article.

## Conflict of Interest Statement

The authors declare that the research was conducted in the absence of any commercial or financial relationships that could be construed as a potential conflict of interest.
